# Statistical learning for turboshaft helicopter accidents using logistic regression

**DOI:** 10.1371/journal.pone.0227334

**Published:** 2020-01-13

**Authors:** Rachmat Subagia, Joseph Homer Saleh, Jared S. Churchwell, Katherine S. Zhang

**Affiliations:** School of Aerospace Engineering, Georgia Institute of Technology, Atlanta, Georgia, United States of America; Tongii University, CHINA

## Abstract

The objective of this work is to advance the understanding of helicopter accidents by examining and quantifying the association between helicopter-specific configurations (number of main rotor blades, number of engines, rotor diameter, and takeoff weight) and the likelihood of accidents. We leverage a dataset of 8,338 turboshaft helicopters in the U.S. civil fleet and 825 accidents from 2005 to 2015. We use the dataset to develop a logistic regression model using the method of purposeful selection, which we exploit for inferential purposes and highlight the novel insights it reveals. For example, one important question for the design and acquisition of helicopters is whether twin-engine turboshaft helicopters exhibit a smaller likelihood of accidents than their single-engine counterparts, all else being equal. The evidence-based result we derive indicates that the answer is contingent on other covariates, and that a tipping point exists in terms of the rotor diameter beyond which the likelihood of accidents of twin-engines is higher (worse) than that of their single-engine counterparts. Another important result derived here is the association between the number of main rotor blades and likelihood of accidents. We found that for single-engine turboshaft helicopters, the four-bladed ones are associated with the lowest likelihood of accidents. We also identified a clear coupling between the number of engines and the rotor diameter in terms of likelihood of accidents. In summary, we establish important relationships between the different helicopter configurations here considered and the likelihood of accident, but these are associations, not causal in nature. The causal pathway, if it exists, may be confounded or mediated by other variables not accounted for here. The results provided here lend themselves to a rich set of interpretive possibilities, and because of their significant safety implications they deserve careful attention from the rotorcraft community.

## 1. Introduction

The work is part of a larger effort whose objective is to provide a better understanding of helicopter accidents, and to identify important areas for different stakeholders in the rotorcraft community to focus their attention and resources on for accident prevention. The end-objective is to contribute toward improving the safety track record of helicopters, and ultimately to reduce the burden of injuries, fatalities, and financial losses due to helicopter accidents.

Rotorcraft fulfill a wide range of functions, and they have become indispensable for many civilian and military applications, from medical evacuation, to delivery of humanitarian aid, law enforcement, and a whole slew of commercial and military applications. This mission flexibility is enabled by the fundamental design feature of these craft, a *rotating wing*, which, in turn, is responsible for a host of challenges and complexities in the design, operation, and maintenance of these systems. These complexities also likely have an imprint on the poor safety track record of helicopters. For context, the fatal accident rate of civilian helicopters in the U.S. has shown little or no progress over the last decade, and it is about 17 times higher than fatal accident rates of passenger cars. Over 120 helicopter accidents occur every year, with several hundred injuries and fatalities, and a few hundred million dollars in yearly losses (details in Churchwell et al. [1], and Saleh et al. [2]). These issues are cursorily noted to motivate the need for more research into helicopter accidents and for more vigorous safety intervention efforts by the regulators and other stakeholders in this industry.

Helicopter accidents have generally not received the same level of attention or thoroughness in the safety literature as accidents in other sectors such as chemical, oil and gas, or airline industries. Studies related to the helicopter safety have typically focused on a specific mission type or particular geographic area of operations. SINTEF, for example, examined trends and risk reduction factors for helicopters in the Norwegian oil and gas operations in the North Sea [3, 4]. These studies were the basis for a number of other investigations for similar helicopter missions and within the same geographic area [5–7]. Other studies have examined helicopter accidents in different areas of operations. Atkinson and Irving [8], for example, examined helicopter accidents in the UK. The authors found that light helicopters (weight less than 2,730 kg) had a significantly higher accident rate than their heavier counterparts; and that about 60% of all accidents were due to pilot errors. Another important study of helicopter safety was carried out by the European Helicopter Safety Team (EHEST) [9]. The organization, re-established as the European Safety Promotion Network Rotorcraft (ESPN-R) since 2017, examined helicopter accidents in Europe and provided recommendation of areas of improvement, including flight operations, and regulatory provisions. Accidents of helicopter medical emergency services (HEMS), another example of mission type, have received some attention in both the mainstream media and the technical literature. The National Transportation Safety Board (NTSB), for instance, HEMS accidents in 1988, and again in 2006 [10, 11]. The Board identified several deficiencies in EMS operations and made a number of recommendations to improve their safety track record, in particular better flight risk assessment and planning. Other researchers also examined medical helicopter accidents in the United States [12, 13] and found that there was a steady increase in the number of medical helicopter accidents during over time. Such findings, however, have to be interpreted cautiously because the lack of exposure data in these studies (the absence of denominator for calculating accident rates, such as total number of medical helicopters in operation or their total flight hours). A more extensive literature review can be found in the related articles by the authors [1, 2].

In this work, we seek to further advance the understanding of helicopter accidents and their association with a set of attributes or covariates. Our objectives are both methodological in nature and content-specific related to helicopter configurations. More precisely, our first objective is to demonstrate the application of logistic regression to modeling helicopter accidents. We detail in the next subsections our model-building process, the use of the developed model for inferential purposes, and the novel insights this statistical tool can provide. Our second objective is to identify and quantify the association, if any, between helicopter-specific configurations or attributes and their likelihood of accidents.

Once our logistic model is built and its goodness of fit is verified, we leverage it to investigate a set of research questions subsumed under this second objective. For example, one important question for the design and acquisition of helicopters is whether or not twin-engine helicopters exhibit a smaller likelihood of accidents than their single-engine counterparts, all else being equal. Intuition may suggest that this is likely the case. There are important consequences to this in terms of regulatory pressures, for example, in phasing out single-engine helicopters in favor of their twin counterparts. As a result, this question deserves careful attention beyond the common-sense expectation. The evidence-based answer, we will show, is more nuanced than the expected intuitive answer, that it is contingent on other covariates, and that a tipping point exists in terms of rotor blade diameter beyond which the likelihood of accidents of twin-engines is worse (higher) than that of their single-engine counterparts. We investigate two other inferential questions under this second objective: Do turboshaft helicopters with different number of main rotor blades have different likelihood of accidents, and if so, are these differences statistically significant? Is it better, worse, or irrelevant from a safety perspective to increase the main rotor diameter, all else being equal? The answer to the latter question can help inform design choices, for example, if wiggle room exists in the selection of rotor diameter beyond the performance, structural, and aerodynamic considerations.

We address these two objectives and provide the answers to these questions in this work. The answers lend themselves to a rich set of interpretive possibilities, and they deserve careful attention from the rotorcraft community. There remainder of this work is organized as follows. Section 2 discusses the data and methods used hereafter. Section 3 develops the logistic model and leverages it for inferential purposes. The limitations of this study are also discussed in Section 3. Section 4 concludes this work.

## 2. Data and methods

The present work utilizes publicly available data of civil helicopters from three sources: (1) the Federal Aviation Administration (FAA) for registration records; (2) the National Transportation Safety board (NTSB) for accident records; and (3) helicopter manufacturers for technical details available online in the public domain. The time frame for the data extends from January 2005 to December 2015. We excluded data related to gyrocopters, experimental and homebuilt helicopters since they are subject to different airworthiness specifications as well as different regulations regarding their maintenance and usage. We also excluded data recorded in territories and geographic regions within the domain of the FAA and NTSB but not classified as states, such as Virgin Islands and Puerto Rico. The reason for this was that the data from these regions were sparse and oftentimes inadequately recorded. We only include in our analysis adverse events formally classified as *accident*s. The NTSB defines accident as an occurrence: (1) associated with the operation of an aircraft that takes place between the time any person boards the aircraft with the intention of flight and all such persons have disembarked; (2) in which any person suffers death or serious injury; (3) in which the aircraft receives substantial damage. *Incidents*, minor adverse events that affect or could affect the safety of operations and which result in light or no damage, are not considered in this analysis. Their examination offers nonetheless useful learning opportunities and are left as fruitful venue for future work. The application of these exclusion filters resulted in a dataset with 13,055 helicopters and 1,572 accidents over the 11-year timespan of study.

For each helicopter, the dataset includes its identification (ID) number, whether it experienced an accident or not—this is the response variable in our analysis—and the following attributes or potential covariates: (1) the number of main rotor blades; (2) the number of engines; (3) the type of engines; (4) the diameter of main rotor blades (rotor diameter); and (5) the maximum takeoff weight (MTOW).

We treat MTOW as continuous variable instead of classifying it into discrete light/medium/heavy bins for two reasons. First, different agencies set different thresholds for helicopter weight category. The FAA, for example, does not categorize helicopters as light/medium/heavy, but as normal or transport helicopters based on MTOW: transport helicopters are those with MTOW greater than 7,000 pounds (3,175 kg). However, the National Interagency Fire Center (NIFC) does categorize helicopters into three bins: light helicopters with certified gross weight less than 6,000 pounds (2,722 kg), medium helicopters between 6,000 and 12,500 pounds (2,722 and 5,670 kg), and heavy helicopters with certified gross weight exceeding 12,500 pounds (5,670 kg). Second, from a statistical point of view, several studies [14, 15] have demonstrated that categorizing continuous variables leads to the loss of important information and increase in type I error. The categorization of continuous variable can also conceal possible nonlinearity in the relation between the variable and outcome, in addition to the fact that individual helicopters close to but on the opposite side of the thresholds are classified as being different rather than similar.

Table 1 provides a four-level frequency summary of our dataset. It shows, for example, that there is only one data point for a twin-engine helicopter with reciprocating engines. Furthermore, Table 1 shows that there are very few or no helicopters with a single reciprocating engine in the 5- and 6-blade categories. This has an important implication: there is insufficient data to conduct statistical analysis using the entire dataset without encountering numerical problems (ill-conditioned or singular matrix). For this reason, we only focus in this work on turboshaft helicopters. Helicopters equipped with reciprocating engines, or only single engines, will be examined in future work. During the 11-year period of interest in this study, there were 8,338 turboshaft helicopters (64% of the original dataset) and 825 accidents.

**Table 1:** Four-level frequency summary of civil helicopter dataset in the U.S. from 2005 to 2015.

Accident and Number of main rotor blades	Number of engines and Engine type			
	single engine		twin engine	
	Reciprocating	Turboshaft*	Reciprocating	Turboshaft*
No				
2	3,070	2,452	0	159
3	843	1,256	0	47
4	54	1,330	0	1,301
5	0	531	0	255
6	2**	36	1**	146
Yes				
2	568	298	0	15
3	174	171	0	5
4	5	110	0	81
5	0	121	0	9
6	0	8	0	7

* The dataset used for present work is shaded grey.

** These helicopters were registered during the time window of study regardless whether or not they are actively flown.

To examine turboshaft helicopter accidents, we use logistic regression with this turboshaft dataset to construct a mathematical model for inferential purposes given a set of covariates (or configuration) **x**. This is the conditional probability that an accident occurs given a configuration captured in the vector **x**, Pr(accident = 1|**x**), which in general take the following form:

$$Pr(accident=1|x)\equiv \pi (\beta ,x)=\frac{e^{\beta_{0}+\beta_{1}x_{1}+\beta_{2}x_{2}+\cdots }}{1+\;e^{\beta_{0}+\beta_{1}x_{1}+\beta_{2}x_{2}+\cdots }}$$ (1)

The unknowns in Eq (1) are the coefficients β_*i*_ or the vector **β** whose components are these individual coefficients, and the form of the power in the exponent. The observations are assumed to be independent and identically distributed. To fit this model to the observed data, logistic regression uses maximum likelihood estimation to determine the best estimated coefficients:

$${\text{max}}_{\beta }\prod_{i=1}^{N_{obs.}}{\pi (\beta ,x_{i})}^{{accident}_{i}}{\left[1-\pi (\beta ,x_{i})\right]}^{\left(1-{accident}_{i}\right)}$$ (2)

A brief explanation of what the odds and odds ratio are warranted since these measures of association are central to logistic analysis, and they may not be as familiar to an engineering audience as other measures of association such as risk ratio. In our study, the odds of an accident is defined as the ratio of the probability that an accident occurs to the probability that the accident does not occur for a given configuration **x**:

$$odds\equiv \frac{\text{Pr}\left(accident=1|x\right)}{\text{Pr}\left(accident=0|x\right)}=\frac{\pi (\beta ,x)}{1-\pi (\beta ,x)}$$ (3)

By taking the log of the odds of accident and substituting Eq (1) to this expression, we obtain what is defined as the logit transformation g(**x**), which is linear in its parameters:

$$g(x)\equiv logit[\pi (\beta ,x)]\equiv \text{ln}\left[odds\right]=\text{ln}\left[\frac{\pi (\beta ,x)}{1-\pi (\beta ,x)}\right]=\beta_{0}+\beta_{1}x_{1}+\beta_{2}x_{2}+\cdots$$ (4)

A particularly meaningful interpretation arises when we exponentiate the coefficient associated with a variable in Eq (4). Consider, for example, dichotomous variable *x*_2_ whose value is either 0 and 1, and suppose that this value represents single and twin engine respectively. Therefore, the ratio of the odds of accidents for twin engine (*x*_2_ = 1) to the odds of accidents for single engine (*x*_2_ = 0), all else being equal, is given by the odds ratio (OR):

$$OR=\frac{\frac{\pi (x_{2}=1)}{\left[1-\pi (x_{2}=1)\right]}}{\frac{\pi (x_{2}=0)}{\left[1-\pi (x_{2}=0)\right]}}=e^{\beta_{2}}$$ (5)

Similar process also applies to other variables. A single coefficient is therefore a measure of the log of the odds ratio for different value of the associated variable in logistic regression (this is an adjusted odds ratio, which controls for all the other covariates *x*_*i*≠2_). This is a powerful result, and it accounts in part for the extensive adoption of logistic regression in many fields to model categorical variables. A more nuanced interpretation of the coefficients, however, is required when statistical interaction is present in the model. This will be carefully examined in the next section.

In the analysis that follows, we lumped the two-bladed and three-bladed turboshaft helicopters into one category. The reason for this was because we found that these two types of helicopters exhibited the same odds of accidents. Furthermore, we found that the models with 2-bladed and 3-bladed helicopters as separate groups on the one hand, and the model with the two as one group on the other hand generated identical results. Therefore, the categorization of the number of main rotor blades used in this study, and the associated design (dummy) variables are shown in Table 2.

**Table 2:** The dummy variables and their value for each category used in this analysis.

Category of number of blades	Dummy variables			Category of number of engines	Dummy variable
	*x*_1,1_ (N_BLADES1)	*x*_1,2_ (N_BLADES2)	*x*_1,3_ (N_BLADES3)		*x*_2_ (N_ENGINES)
2B-and-3B	0	0	0	Single	0
4B	1	0	0	Twin	1
5B	0	1	0		
6B	0	0	1		

**Fig 1:**
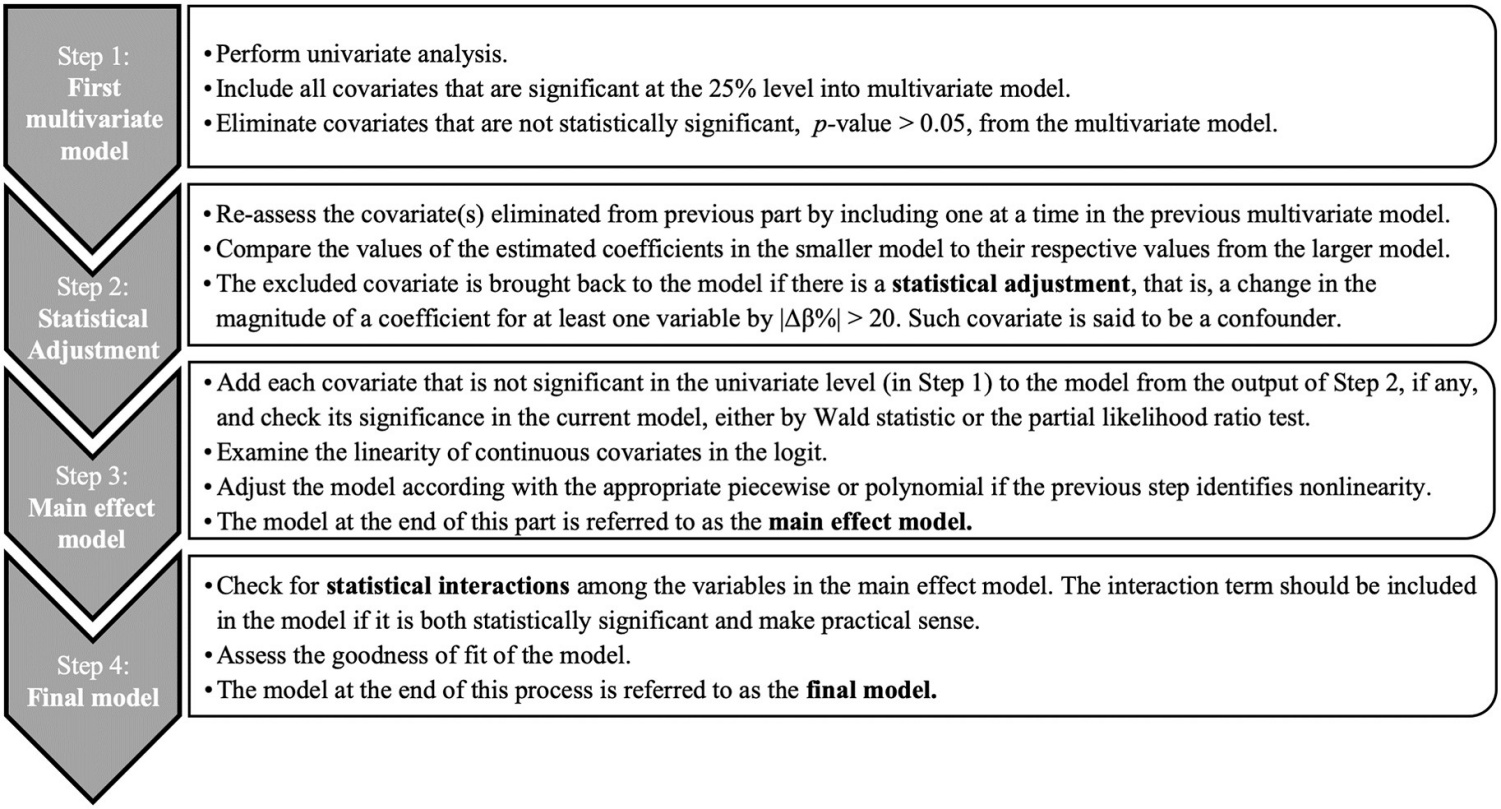
Model-building strategy using method of purposeful selection developed by Hosmer et al. [16].

To determine the best linear (in the parameters) form of the logistic regression model as well as the associated coefficients, we adopted the method of purposeful selection in logistic regression as our model-building strategy (developed by Hosmer et al. [16]). The method begins with a univariate analysis of each variable to assess the relevance, or lack thereof, of each candidate covariate for the response variable, here the likelihood of accident. It is then extended to multivariate logistic regression with all previously identified statistically significant covariates included, and further down-selection is applied (details in Fig 1 and next section). The method then diagnoses for possible confounders and assesses the scale (linearity) of the continuous variables. It then investigates the presence of statistical interactions to account for effect modifier(s), as this could significantly affect the odds ratio of accidents for the associated covariates and, hence, its model fitness. Once the final model is developed, we examine its goodness-of-fit, and we make extensive use of it for inference purposes in the next section. Throughout this study, the logistic regression analysis was performed using Stata version 15.1 software (Stata Corporation, College Station, TX).

## 3. Turboshaft helicopter accidents: Statistical learning, result, and discussion

We begin this section by executing on the model-building strategy discussed previously to develop the logistic model of turboshaft helicopter accidents. We then leverage this model for inferential purposes and determine the effect of varying each covariate (e.g., number of main rotor blades, number of engines) on the likelihood of accidents. Finally, we discuss the limitations of this analysis.

### 3.1 Logistic model building

The following discussion explains how we converged on the final logistic model for turboshaft helicopter accidents. The reader only interested in the end result can skip the developments in Section 3.1.1 and 3.1.2 and jump ahead to the final model expressed in Eq (7).

#### 3.1.1 From univariate to multivariate models

The first step in the method of purposeful selection is to fit a univariate logistic regression model for each covariate (one at a time). The objective of this step is to examine the significance of the covariate to the model at the 25% level. The results of this analysis are shown in Table 3. Note that in this table, each row presents the results for the estimated regression coefficients from a model containing only that single covariate. The statistical significance of the estimated coefficient or crude odds ratio is examined using the Wald test and the associated *p*-value, that is, the likelihood that the null hypothesis *H*_0_ (the odds of accidents of two types of helicopters are equal) is true given the data. For example, when considering the number of main rotor blades (N_BLADES) as the only covariate, the penultimate column in Table 3 shows a crude odds ratio of 0.58, that is, the odds of accident of four-bladed turboshaft helicopters is 0.58 times the odds of accident of two- or three-bladed turboshaft helicopters. Similar interpretation applies to the univariate model with the number of engines (N_ENGINES) as the only covariate. The result in Table 3 shows a crude odds ratio of 0.49, that is, the odds of accidents of turboshaft helicopters with twin engines is nearly one-half the odds of accidents of turboshaft helicopters with a single engine.

**Table 3:** Results of fitting univariate logistic regression model in the turboshaft helicopter data (one covariate at a time).

Variable	Estimated Coeff. ($\hat{\beta }$)	Standard Error ($\hat{SE}$)	*z*	*p*-value	$\hat{OR}$	95% CI of $\hat{OR}$
N_BLADES						
N_BLADES1	-0.543	0.089	-6.10	<0.001	0.58	0.49–0.69
N_BLADES2	0.281	0.106	2.64	0.008	1.32	1.08–1.63
N_BLADES3	-0.416	0.273	-1.52	0.127	0.66	0.39–1.13
N_ENGINES	-0.723	0.103	-7.00	<0.001	0.49	0.40–0.59
ROTORDIA	-0.061	0.006	-9.62	<0.001	0.94	0.93–0.95
MTOW	-0.096	0.012	-8.25	<0.001	0.91*	0.89–0.93

OR, Odds ratio; CI, Confidence Interval

* OR for 1,000 pounds (455 kg) increase in MTOW

The interpretation of the logistic model for continuous covariate is different from categorical ones. As an example, the estimated odds ratio in the univariate model containing only the rotor diameter (ROTORDIA) in Table 3 is interpreted to mean that for every increase of 1 ft (0.305 m) in rotor diameter, the odds of accidents changes by 0.94 times.

The results in Table 3 should not be used for inferential purposes as we have not yet considered all the covariates together nor have we accounted for the possibility that each model is confounded by other covariates. For our purposes given our model-building strategy (Fig 1), all the covariates, when examined independently (Table 3), are significant below the 25% level. Consequently, we include them all in our first multivariate model. This completes Step 1 in Fig 1.

The suite of building multivariate model is not straightforward, and several intermediate results are embedded in the process. To facilitate its understanding, we decompose the multivariate model development into three major steps shown in Fig 1: linear multivariate model (Step 2); main effect model (Step 3); and final model (Step 4). The results from Step 2 are discussed shortly, and the two remaining steps are addressed in the next subsections.

The linear multivariate model refers to the model from the output of Step 2 in Fig 1, where we evaluate for confounders, under the assumption that the continuous covariates are linear in the logit. In intermediate results not shown here, the presence of N_ENGINES in the model brings statistical adjustment to N_BLADES, and MTOW brings statistical adjustment to both N_BLADES and ROTORDIA. The implication of this fact is that, although the estimated coefficients for N_ENGINES and MTOW are not statistically significant in the multivariate model, their presence brings notable changes to the estimated coefficient for N_BLADES and ROTORDIA, and hence, should be included in the model. Therefore, the linear multivariate model contains all four covariates: N_BLADES, N_ENGINES, ROTORDIA and MTOW.

#### 3.1.2 Assessment of linearity assumption for continuous covariates

The model development is far from over at the end of the previous step. The next step of the multivariate model development is referred to as the main effect model, and it is obtained after we investigate the linearity assumption of the continuous covariates involved in the previous step, i.e., rotor diameter (ROTORDIA) and MTOW. To do this, we present three different methods for comparison: smoothed scatter plot (lowess smooth), fractional polynomials, and linear spline functions. The lowess smoothing is a nonparametric approach that provides some indication about the relationship between the outcome (here, accidents) and the continuous covariate by computing the weighted average of the outcome from the subsets of bandwidth *k* × *N* observations. The default bandwidth *k* = 0.8 is used in this analysis. Note that the weighted average of the outcome is often expressed in the logit form using similar transformation as in Eq (4) for logistic regression. Therefore, if the lowess smoothing approach shows linear in the logit, then the continuous covariate is likely to be linear. Otherwise, if nonlinearity is detected, we would need to use an analytical approach to fit a parametric function that would satisfactorily capture the displayed nonlinearity; this is addressed by the two remaining methods discussed next.

The idea of fractional polynomials as developed by Royston and Altman [17] is to determine the number of terms and their corresponding powers for the continuous covariates in the logit that best fits the data. This is achieved first by an iterative process to search for the best powers for each number of terms (e.g., one-term, two-term continuous covariate model), then comparing them one another as well as with the model in which the continuous covariate is linear (details can be found in [16, 18]). The third method used here is spline function, which takes the idea of the draftsman’s spline to fit a series of smooth curves joined at specified points called knots. In this work, we only consider linear spline with one knot for which the location is determined based on the behavior depicted in the lowess smooth.

**Fig 2:**
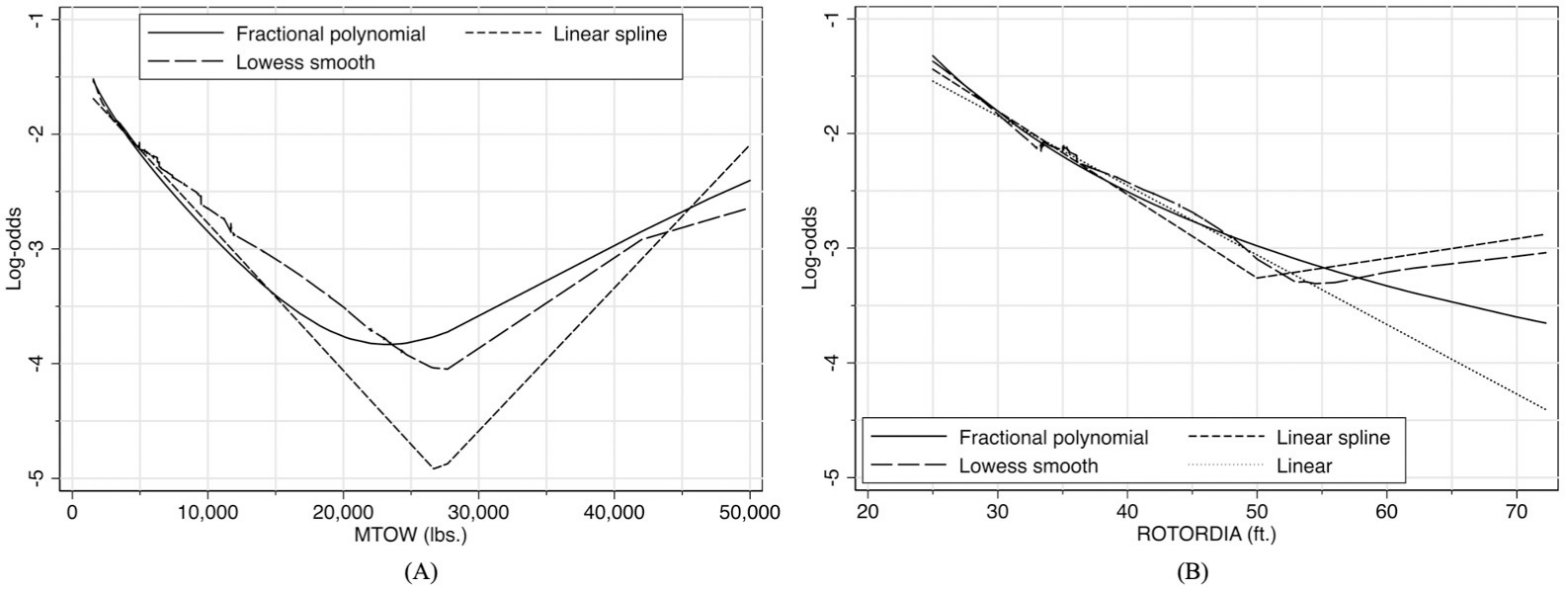
The examination of linearity assumption in the logit for continuous covariate among turboshaft helicopters. (A) two-term fractional polynomial (powers .5 and 1), linear spline with one knot at 27,000 lbs (12,250 kg), and lowess smooth for MTOW; (B) one-term fractional polynomial (power -2), linear spline with one knot at 50 ft (15.24 m), and lowess smooth for rotor diameter.

The application of these three methods for turboshaft helicopter accidents is shown in Fig 2, where the y-axis represents the log-odds of helicopter accidents, g(**x**). In Fig 2A, for example, we see a clear nonlinearity in the log-odds as a function of MTOW, and that the fractional polynomial fits better than the linear spline in capturing the lowess smooth behavior. In addition, in a side analysis not shown here, we calculated the deviance of the model (that is defined as D = -2×log-likelihood) with the two-term fractional polynomial and found it is smaller than that of the model with the linear spline, which further confirms that the fractional polynomial has the better fit to the model nonlinearity of MTOW in the logit.

The result of the scale of the continuous covariate ROTORDIA in Fig 2B raises an interesting dilemma. On the one hand, the one-term fractional polynomial fits the lowess smooth better than the linear spline when the rotor diameter is lower than 53 ft (16.15 m). On the other hand, the linear spline is able to capture the change in slope as the lowess smooth. However, by examining the source data, we found that the change in slope of the lowess smooth is due to only three accidents that occurred to helicopters with rotor diameter of 72 ft (21.95 m). Therefore, the exclusion of these helicopters and those with similar configurations, which is less than 0.1% of the dataset, leads to a linear behavior of ROTORDIA in the logit without measurably changing the estimated coefficient in the model. For this reason, we decided that the linearity assumption for ROTORDIA in the logit is valid. For added precaution and to remain on the conservative side, we propose that the results that follow not be extended beyond rotor diameters larger than 72 ft (21.95 m).

Hence, we conclude that the main effect model contains all four covariates (N_BLADES, N_ENGINES, ROTORDIA, and MTOW) whose logit g(**x**) is linear in ROTORDIA and nonlinear in MTOW with two-term fractional polynomial as described in Fig 2. This is the output of Step 3 in Fig 1. Therefore, the MTOW appears in two terms in the estimated logit of the main effect model as follows:

$$\hat{g}(x)=\cdots +(-0.177)\times {\left(\frac{MTOW}{100}\right)}^{0.5}+0.005\times \left(\frac{MTOW}{100}\right)+\cdots$$ (6)

In an intermediate result not shown here, we found that the estimated coefficients of the main effect model are in general not statistically significant, except for the 4B versus the 2B-and-3B turboshaft helicopters. This situation indicates that the main effect model generates estimated probability of accidents with high standard errors, despite the fact that all of the important covariates are included. This is the main motivation for Step 4 in the model-building strategy: to explore the possibility of statistical interactions between covariates, and in the process to improve the performance of the fitted model. We examine this issue in the next subsection.

#### 3.1.3 Statistical interactions and goodness of fit

The last step in the development of the multivariate model is completed after we examine whether or not statistical interactions between covariates exist and are statistically meaningful, and after checking the goodness-of-fit of the (candidate) final model with all the covariates and their possible interactions.

A statistical interaction describes a situation in which the effect of one covariate on the outcome depends on the state of the second covariate. The presence of the statistical interaction in a model implies that the odds ratio for the covariate that has the interaction must be specified at certain values. To better appreciate this, consider a hypothetical situation in epidemiology for example, where the presence or absence of Coronary Heart Disease (CHD) in a person is influenced by their age and gender. The absence of statistical interaction between these two covariates, age and gender, would indicate that the odds ratio of CHD between two individuals with different ages is independent of their gender (the two curves of the log-odds of CHD as a function of age for male and female are parallel). If there is statistical interaction between age and gender, it means that gender modifies the exposure to CHD as a function of age (and the two log-logit curves are not parallel). In which case, we cannot estimate the odds ratio of CHD between individuals of different ages without specifying their gender. In other words, gender is an effect modifier because it controls the effect of age on the CHD outcome.

In logistic regression, the statistical interaction between two variables is modeled by the inclusion of product terms of the two in the logit. For the method of purposeful selection, the search for relevant statistical interaction in the fitted model begins by the inclusion of each possible pair of interaction to the main effect model one at a time. The statistically significant and practically sensible interaction(s) are then included in the model for further assessment. We tested all possible interactions (not shown here for the sake of brevity) and found that only three interactions are important and statistically meaningful for further assessment. They are the interactions between: the number of main rotor blades with the number of engines; the number of engines with the rotor diameter; and the rotor diameter with the MTOW. Table 4 confirms this conclusion and demonstrates that these statistical interactions significantly improve the fitted model over the previous main effect model (seen here in the increase of the log-likelihood and the *p*-values of the corresponding partial likelihood-ratio test).

**Table 4:** Statistical significance test for the addition of interactions (one at a time) to the main effect model of turboshaft helicopter accidents.

Interaction	Log-likelihood	G	DoF	*p*-value
Main effect model	-2613.9697			
N_BLADES×N_ENGINES	-2609.3536	9.23	3	0.026
N_ENGINES×ROTORDIA	-2610.1105	7.72	1	0.005
ROTORDIA×MTOW	-2608.6444	10.65	2	0.005

DoF, Degree of Freedom.

The inclusion of these three statistical interactions generates the model in Table 5. Note that an interaction is included in the model as long as at least one component is statistically significant [16]. In Table 5, we see this situation with the interaction between N_BLADES2×N_ENGINES which is statistically significant (*p*-value < 0.05). Thus, the interaction between the number of main rotor blades and the number of engines is included in the model despite the fact that the other two interaction components are not statistically significant. Next, we assess the model adequacy and its fitness before using it for inferential purposes.

**Table 5:** Final model of turboshaft helicopter accidents with statistical interactions.

	x	Estimated Coeff. ($\hat{\beta }$)	Standard Error ($\hat{SE}$)	*z*	*p*-value	95% Confidence Interval	
N_BLADES							
N_BLADES1	*x*_1,1_	-0.390	0.118	-3.29	0.001	-0.622	-0.158
N_BLADES2	*x*_1,2_	0.792	0.245	3.24	0.001	0.313	1.272
N_BLADES3	*x*_1,3_	0.879	0.447	1.97	0.049	0.003	1.755
N_ENGINES	*x*_2_	-3.179	0.937	-3.39	0.001	-5.016	-1.343
ROTORDIA	*x*_3_	0.264	0.084	3.13	0.002	0.099	0.430
MTOW1*	*x*_4_	1.836	0.470	3.91	<0.001	0.915	2.758
MTOW2**	*x*_5_	-0.081	0.022	-3.66	<0.001	-0.124	-0.038
N_BLADES×N_ENGINES							
N_BLADES1×N_ENGINES	*x*_6,1_	0.147	0.304	0.48	0.628	-0.448	0.742
N_BLADES2×N_ENGINES	*x*_6,2_	-1.265	0.447	-2.83	0.005	-2.142	-0.388
N_BLADES3×N_ENGINES	*x*_6,3_	-1.304	0.845	-1.54	0.123	-2.960	0.351
N_ENGINES×ROTORDIA	*x*_7_	0.083	0.022	3.79	<0.001	0.040	0.126
ROTORDIA×MTOW1*	*x*_8_	-0.050	0.012	-3.99	<0.001	-0.075	-0.025
ROTORDIA×MTOW2**	*x*_9_	0.002	0.0004	4.33	<0.001	0.001	0.003
Constant	(*x*_0_ = 1)	-11.147	2.839	-3.93	<0.001	-16.712	-5.582

* MTOW1 is a scaled MTOW with power 0.5 (MTOW1 = (MTOW/100)^0.5^)

** MTOW2 is a scaled MTOW with linear term (MTOW2 = MTOW/100)

To assess the model fitness, we conduct two different tests: the Hosmer-Lemeshow (H-L) test [16] and the Stukel’s test [19]. The H-L test is conducted by grouping the estimated probability based on percentiles and comparing with the observation data. The goodness-of-fit test using this method obtains that the *p*-value computed from the chi-square distribution with 6 degrees of freedom is 0.3317. Therefore, the null hypothesis *H*_0_ that the probability of accidents in each group between estimated and observation data is similar cannot be rejected. In other words, a comparison of the observed versus expected frequencies in each group shows close agreement, as shown in Table A in S1 Appendix. The Stukel’s test provides a similar conclusion. Hence, we conclude that the model shown in Table 5 is our final model for turboshaft helicopter accidents. The estimated probability of accident and its 95% confidence interval for a given configuration (number of main rotor blades, number of engines, rotor diameter, and MTOW) is written in mathematical form as follows:

$$\hat{Pr}(accident=1|x)=\frac{e^{\hat{g}(x)}}{1+e^{\hat{g}(x)}}\;\text{and}\;{\hat{Pr}}_{95\%\;CI}=\frac{e^{\hat{g}(x)\pm 1.96\times \hat{SE}[\hat{g}(x)]}}{1+e^{\hat{g}(x)\pm 1.96\times \hat{SE}[\hat{g}(x)]}}$$ (7)

where

g^(x)=β^0+∑j=13(β^1,jx1,j+β^6,jx6,j)+∑i=2i≠69β^ixi (8)

and the associated estimated standard error of Eq (8) can be expressed accordingly. The estimated coefficients in the equation are provided in Table 5, and the associated covariance matrix can be found in Table B in S1 Appendix.

### 3.2 Model inference: Adjusted odds ratio and implications

We now begin to unpack the implications contained in the final model developed in the previous subsection. We examine hereafter the odds ratio of accidents by changing one covariate at a time. First, we examine the odds ratio of accidents for turboshaft helicopters with different number of main rotor blades while controlling for all the other covariates (i.e., all else being equal), the reference group being the 2B-and-3B helicopters. Second, we examine the odds ratio of accidents for turboshaft helicopters with single versus twin engines. Third, we examine the odds ratio of accidents for turboshaft helicopters with different rotor diameters. Because of statistical interactions, the results cannot be displayed as single values, but will be presented in either a stratified manner, e.g., separately for single and twin engines for the first analysis, or as a function of one of the continuous covariates for the other two. The following discussion will further clarify these issues.

#### 3.2.1 Adjusted odds ratio for different number of main rotor blades

One of the main motivations for this work was the simple question of whether turboshaft helicopters with different number of main rotor blades exhibited different likelihood of accidents. A more nuanced questions can now be formulated and addressed given our logistic model: do turboshaft helicopters with different number of main rotor blades have different odds of accidents, that is, controlling for all other covariates? And if so, are these differences statistically significant? To investigate these questions, we leverage the final model in Table 5 and Eq (7), and compute the estimated odds ratio of accidents for different number of main rotor blades, where the 2B-and-3B turboshaft helicopters are chosen as the reference group. As an example, the log-odds ratio of accidents for the 4B versus the 2B-and-3B turboshaft helicopters is computed as follows:

$$\begin{array}{c} \text{ln}\left[\hat{OR}(4B,2B\;and\;3B)\right]=\hat{g}(N\_BLADES=4B)-\hat{g}(N\_BLADES=2B\;and\;3B)\\ ={\hat{\beta }}_{1,1}+{\hat{\beta }}_{6,1}\times N\_ENGINES\; \end{array}$$ (9)

Note that the log-odds ratio in Eq (9) depends on another covariate, namely the number of engines due to the statistical interaction between these two. Therefore, to compute the estimated odds ratio of accidents for different number of main rotor blades, we have to specify the number of engines. The corresponding variance of the log-odds ratio can also be computed to provide the 95% confidence interval. For example, the log-odds ratio for the 4B turboshaft helicopters versus their 2B-and-3B counterparts among those with twin engines is $\text{ln}\;[\text{OR}\left(4\text{B},2\text{B-and-}3\text{B}\right)]={\hat{\beta }}_{1,1}+{\hat{\beta }}_{6,1}$. The associated estimated variance is computed as follows:

$$\hat{Var}(\text{ln}\left[\hat{OR}(4B,2B\;and\;3B)\right])=\hat{Var}({\hat{\beta }}_{1,1})+\hat{Var}({\hat{\beta }}_{6,1})+2\times \hat{Cov}({\hat{\beta }}_{1,1},{\hat{\beta }}_{6,1})$$ (10)

**Fig 3:**
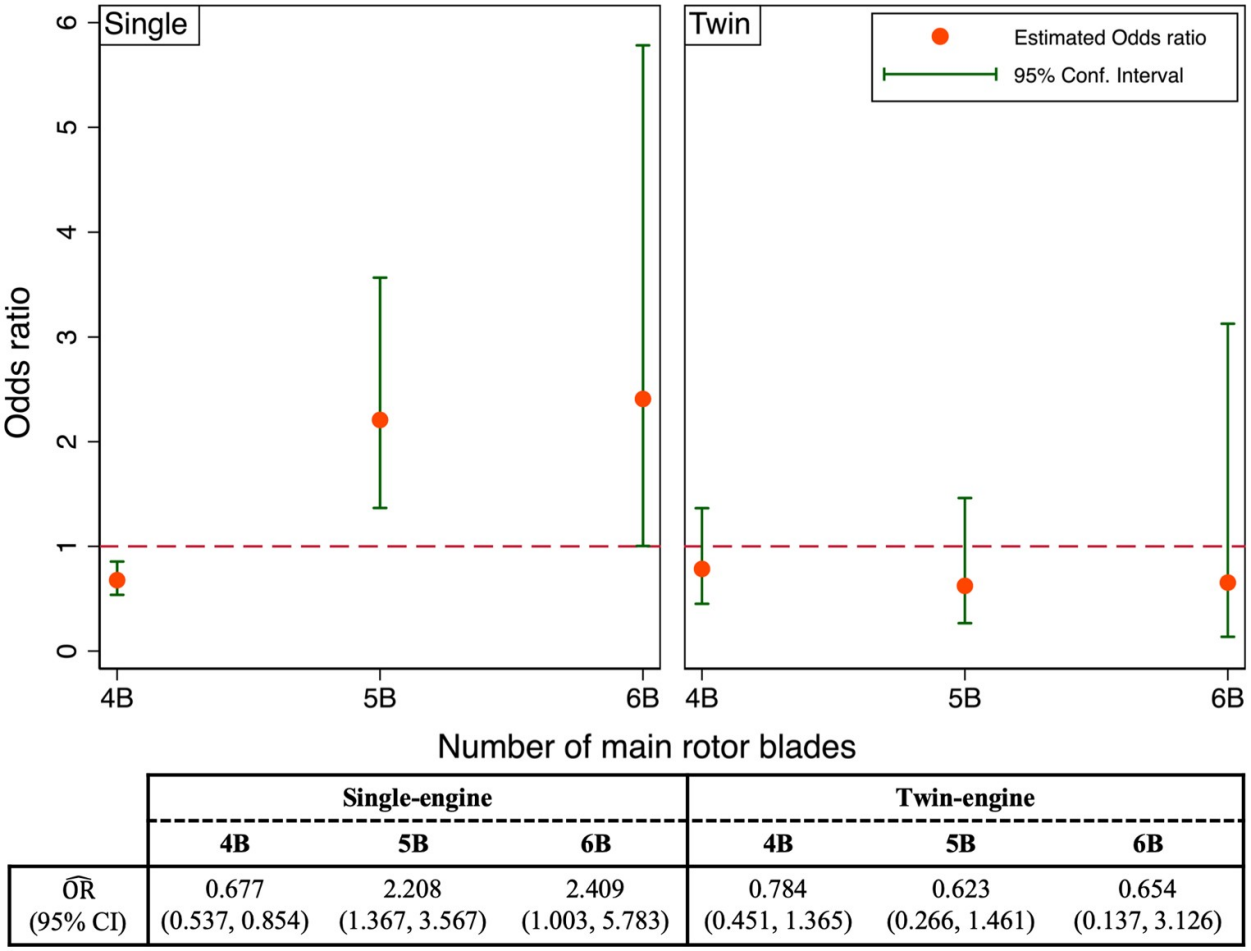
The estimated odds ratio for 4B, 5B, and 6B versus 2B-and-3B turboshaft helicopters.

The covariance matrix is provided in Table B in S1 Appendix. This process is repeated for the single and twin engines, and for the other number of main rotor blades (5B and 6B versus 2B-and-3B). The results are shown in Fig 3.

How to interpret these results? Consider, for example, turboshaft helicopters with single engine (the left panel in Fig 3), the ones equipped with four main rotor blades are associated with approximately 0.677 times the odds of accidents of the reference group (2B-and-3B), all other covariates held constant. In addition, the 95% confidence interval of this odds ratio varies from 0.537 to 0.854, thus indicating that there is a statistically significant difference between the odds of accidents for the single-engine 4B turboshaft helicopters and the 2B-and-3B counterparts. For the single-engine 5B and 6B turboshaft helicopters, their odds ratios of accidents with respect to the reference group (2B-and-3B) are 2.2 and 2.4 respectively. And these differences are statistically significant. These are important findings, and they can help inform design and acquisition choices for helicopter manufacturers and operators. For example, if a single-engine turboshaft helicopter is under consideration for development or acquisition, all else being equal, the four-bladed choice is the best-in-class, and the five- or six-bladed choices are the worst-in-class in terms of their proneness to accidents.

The situation is different for the twin engines. As can be seen in the right panel of Fig 3, the odds of accidents of the 4B, 5B, and 6B turboshaft helicopters are lower than the odds of the reference group, namely the 2B-and-3B ones, and the odds ratios being 0.78, 0.62, and 0.65 respectively. But these differences are not statistically significant since the 95% confidence intervals contain the null value that the odds of the two are the same (OR = 1). The implication of these findings is that safety considerations have no bearing on, or do not help inform the selection of the number of main rotor blades for twin-engine turboshaft helicopters.

#### 3.2.2 Adjusted odds ratio of twin- versus single-engine

Another main motivation for this work was the fundamental question of whether or not twin-engine turboshaft helicopters exhibited a smaller likelihood of accidents than their single-engine counterparts, all else being equal. Common sense and intuition may suggest that this is the case. There are important consequences to this intuition in terms of regulatory pressures, for example, in phasing out single-engine turboshaft helicopters in favor of the twins. As a result, this question deserves careful attention beyond the common-sense expectation. To investigate this question, we leverage again the final model in Table 5 and Eq (7) and compute the estimated odds ratio of accidents for different number of engines, where the single-engine turboshaft helicopters are chosen as the reference group.

The results are more nuanced and involved than what the common-sense answer would have been expected to be. To explain them, we begin by computing the log-odds ratio for twin- versus single-engine turboshaft helicopters as follows:

$$\begin{array}{c} \text{ln}\left[\hat{OR}(twin,single)\right]=\hat{g}(N\_ENGINES=twin)-\hat{g}(N\_ENGINES=single)\\ ={\hat{\beta }}_{2}+{\hat{\beta }}_{6,1}\times x_{1,1}+{\hat{\beta }}_{6,2}\times x_{1,2}+{\hat{\beta }}_{6,3}\times x_{1,3}+{\hat{\beta }}_{7}\times ROTORDIA \end{array}$$ (11)

**Fig 4:**
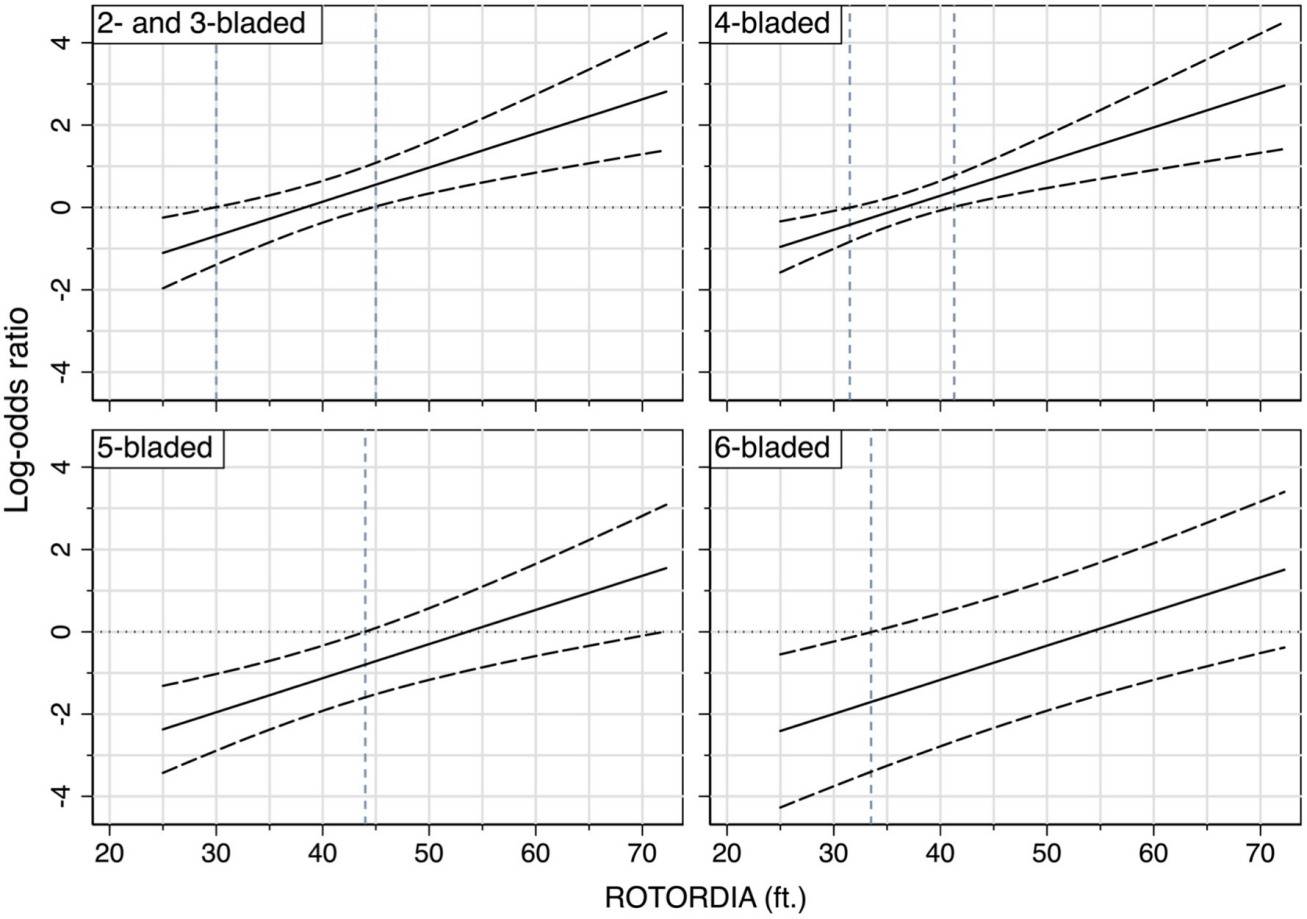
The estimated log-odds ratio for twin- versus single-engine turboshaft helicopters and associated 95% confidence interval.

Note that the log-odds ratio in Eq (11) depends on two covariates, the number of main rotor blades and the rotor diameter, due to statistical interactions between these covariates with the number of engines (as seen in Table 5). In other words, the odds ratio of accidents for different number of engines is controlled by two effect modifiers. The first one, the number of main rotor blades, is a discrete variable, and we isolate its effect by stratifying the analysis and displaying the results separately in four different panels within the same figure. The second one, the rotor diameter, is a continuous variable and we will need to analyze and display the odds ratio as a function of this variable. It will be the x-axis in each of the panels in the figure of these results. Consider for example turboshaft helicopters with four main rotor blades. The log-odds ratio for twin- versus single-engine 4B turboshaft helicopters varies as a function of the rotor diameter:

$$\text{ln}\left[{\hat{OR}}_{4B}(twin,single)\right]={\hat{\beta }}_{2}+{\hat{\beta }}_{6,1}+{\hat{\beta }}_{7}\times ROTORDIA$$ (12)

and the associated estimated variance is computed as follows:

$$\hat{Var}(\text{ln}\left[{\hat{OR}}_{4B}(twin,single)\right])=\hat{Var}({\hat{\beta }}_{2})+\hat{Var}({\hat{\beta }}_{6,1})+{ROTORDIA}^{2}\times \hat{Var}({\hat{\beta }}_{7})+2\times \hat{Cov}({\hat{\beta }}_{2},{\hat{\beta }}_{6,1})+2\times ROTORDIA\times \hat{Cov}({\hat{\beta }}_{2},{\hat{\beta }}_{7})+2\times ROTORDIA\times \hat{Cov}({\hat{\beta }}_{6,1},{\hat{\beta }}_{7})$$ (13)

where the values of the parameters can be found in Table B in S1 Appendix. This process is repeated for the other number of main rotor blades, and we generate the log-odds ratio as well as its associated 95% confidence interval for different values of the rotor diameter as shown in Fig 4. In this figure, we show the estimated log-odds ratio in place of the odds ratio for visual clarity. The estimated odds ratio is obtained by exponentiating these values, which is provided in Table C in S1 Appendix. The zero value in the log-odds ratio corresponds to the null value, OR = 1, where the twin-engine and single-engine turboshaft helicopters have the same odds of accidents. Whereas the negative value in the log-odds ratio corresponds to OR < 1, and it indicates that twin-engine turboshaft helicopters have lower odds of accidents than those with single engine, and vice versa for the positive value of the log-odds, which corresponds to OR > 1.

The results are provided in Fig 4, and they require special attention and careful explanation. Consider for example the upper-right corner panel displaying the results for the 4B turboshaft helicopters. The panel displays three fundamental features to the results: (1) the odds ratio (twin- versus single-engine) of accidents increases (worsens) as the rotor diameter increases; (2) there exists a tipping point beyond which this odds ratio becomes greater than 1 (or the log-odds > 0), which indicates that the twin-engine turboshaft helicopters have a higher propensity to accidents than the single-engine ones, all else being equal; and (3) there are regions within the range of the rotor diameter below or above which the differences in the odds are statistically significant. We explain these in detail in the case of the 4B turboshaft helicopters.

We begin with the tipping point in the odds ratio or log-odds ratio since this delineates two distinctive regions: for a rotor diameter of 37 ft (11.27 m), the odds ratio between twin- versus single-engines is 1, which indicates that there is no advantage to having two engines over a single engine in a four-bladed turboshaft helicopter. Below this rotor diameter threshold, the twin-engine turboshaft helicopters have a lower propensity to accidents than the single-engine counterparts since they exhibit a smaller odds ratio. Above this rotor diameter threshold, the twin engines exhibit a higher propensity to accidents than their single-engine counterparts, all else being equal. To further clarify and emphasize this counter-intuitive result, it states that if the main rotor diameter is larger than 37 ft (11.27 m) on a four-bladed turboshaft helicopter, the twin engines are more prone to accidents than its single-engine counterpart.

Furthermore, the vertical dashed lines in the figure delineates the threshold(s) for which the differences in the odds of accidents for the twin- versus single-engine turboshaft helicopters are statistically significant. For example, in the upper-right corner panel (4B), we see that for rotor diameters below 31.75 ft (9.68 m), the upper bound of the 95% confidence interval is smaller than 1, which indicates there is a safety advantage of twin-engine turboshaft helicopters over their single-engine counterparts. We also see that for rotor diameters above 42 ft (12.80 m), the lower bound of the 95% confidence interval is larger than 1, which indicates a safety advantage of single-engine turboshaft helicopters over their twin-engine counterparts. For rotor diameters between these dashed lines boundaries, from 31.75 ft (9.68 m) to 42 ft (12.80 m), the differences are not statistically significant, and we cannot reject the null hypothesis that a twin-engine turboshaft helicopter offers no safety advantage over a single-engine counterpart, all else being equal. The interpretation of the results in the other panels is similar to the 4B ones. We simply note for instance the small range of rotor diameter below 35 ft (10.67 m), the twin-engine turboshaft helicopters offer a safety advantage over their single-engine counterparts for the 6B. Above that, there is no safety advantage to add another engine.

The causal basis for this intriguing result cannot be resolved with a statistical analysis such as this one, but the data and the modeling clearly demonstrate a coupling between the number of engines and the rotor diameter in terms of safety implications. While this deserves careful attention and further analysis from a structural perspective for example, there are some meaningful implications that can help inform the design of future helicopters.

#### 3.2.3 Adjusted odds ratio for increasing rotor diameter

Given the previous results, we decided to further examine the effects of increasing rotor diameter in isolation of the other covariates. More specifically, we sought to analyze the odds ratio of turboshaft helicopter accidents when the rotor diameter is increased, all else held constant. The general question we seek to address is the following: all else being equal, are we better off increasing a helicopter’s rotor diameter to reduce its odds of accidents, or not? The answer can help inform design choices, for example, when wiggle room exists in the selection of rotor diameter beyond the purely structural and aerodynamic considerations. In other words, the answers can infuse safety considerations in addition to the traditional requirements in the down-selection process of the rotorcraft design.

Unlike the question in the previous subsection, there is no common-sense or intuitive (wrong) answer to this question. We leverage the final model in Table 5 and Eq (7) to address this question. We begin by computing the log-odds ratio for increasing rotor diameter, from *a* to *b* ft., all other covariates held constant:

$$\begin{array}{c} \text{ln}\left[\hat{OR}(b,\;a)\right]=\hat{g}(ROTORDIA=b)-\hat{g}(ROTORDIA=a)\\ ={\hat{\beta }}_{3}(b-a)+{\hat{\beta }}_{7}(b-a)\times N\_ENGINES+{\hat{\beta }}_{8}(b-a)\times {MTOW2}^{0.5}+{\hat{\beta }}_{9}(b-a)\times MTOW2 \end{array}$$ (14)

The reference group in this case is the helicopter with the shorter rotor diameter *a*, and we examine the odds ratio of a similar helicopter in all other aspects except a larger rotor diameter *b*. Note that the log-odds ratio in Eq (14) depends on two covariates, the number of engines and the MTOW, and it is independent of the number of main rotor blades. In other words, the odds ratio for increasing rotor diameter is controlled by these two effect modifiers. The first one, the number of engines is a discrete variable, and we isolate its effect by stratifying the analysis and displaying the results separately in two different panels within the same figure. The second one, MTOW, is a continuous variable and we will need to analyze and display the adjusted odds ratio as a function of this variable. It will be the x-axis in each of the panels in the figure of these results.

Consider, for example, single-engine turboshaft helicopters, their log-odds ratio for an increase of 1 ft (0.305 m) in rotor diameter is expressed as follows:

$$\text{ln}\left[{\hat{OR}}_{single}(1ft.)\right]={\hat{\beta }}_{3}+{\hat{\beta }}_{8}\times {MTOW2}^{0.5}+{\hat{\beta }}_{9}\times MTOW2$$ (15)

and the associated estimated variance is

$$\hat{Var}(\text{ln}\left[{\hat{OR}}_{single}(1ft.)\right])=\hat{Var}({\hat{\beta }}_{3})+MTOW2\times \hat{Var}({\hat{\beta }}_{9})+{MTOW2}^{2}\times \hat{Var}({\hat{\beta }}_{9})+2\times {MTOW2}^{0.5}\times \hat{Cov}({\hat{\beta }}_{3},{\hat{\beta }}_{8})+2\times MTOW2\times \hat{Cov}({\hat{\beta }}_{3},{\hat{\beta }}_{9})+2\times {MTOW2}^{1.5}\times \hat{Cov}({\hat{\beta }}_{8},{\hat{\beta }}_{9})$$ (16)

**Fig 5:**
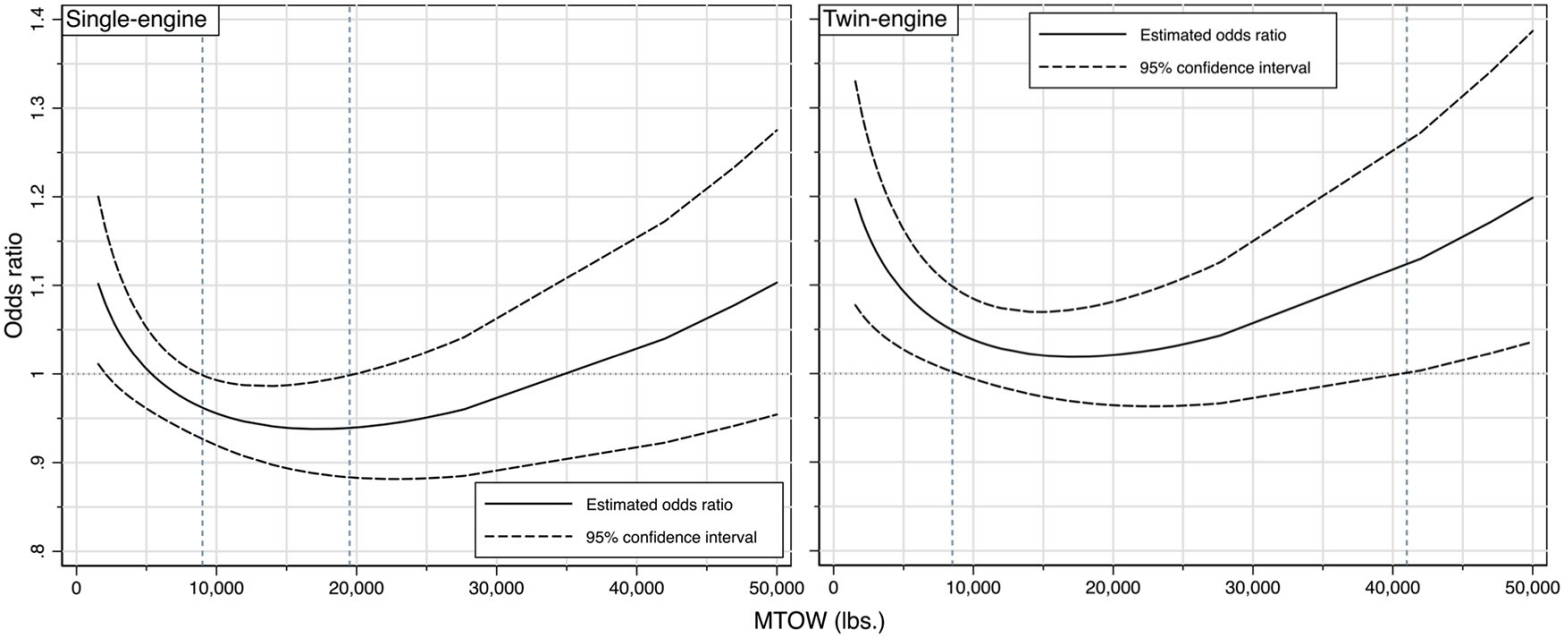
The odds ratio for 1 ft. (0.305 m) increase in rotor diameter among turboshaft helicopters.

Similar calculations are repeated for the twin-engine turboshaft helicopters. By exponentiating the log-odds ratio, we generate the estimated odds ratio for an increase of 1 ft (0.305 m) in rotor diameter as a function of the MTOW and its associated 95% confidence interval. The results are provided in Fig 5, and they require special attention and careful explanation.

Consider for example the right panel, which displays the odds ratio of accidents of twin-engine turboshaft helicopters for an increase in the main rotor diameter by 1 ft (0.305 m). We see again the tipping points and ranges of the MTOW where increasing the rotor diameter provides either a safety advantage or penalty in terms of decreasing or increasing the odds ratio of accidents. More specifically, we see that for the twin engines, the odds ratio of increasing the rotor diameter is always greater than 1, which indicates an increase in the propensity to accidents along with an increase in rotor diameter. Moreover, this increase is statistically significant below the MTOW threshold of 8,500 lbs (3,856 kg) and above 41,000 lbs (18,597 kg), as shown by the lower bound of the 95% confidence interval and the dashed line boundaries. A casual summary of this result can be stated as follows: for twin-engine turboshaft helicopters, it is better to keep the rotor diameter as short as required by other structural and aerodynamic considerations and not any longer.

The results for the single-engine turboshaft helicopters, however, are different. Notice for example in the left panel that for MTOW between 9,000 lbs (4,082 kg) and 19,500 lbs (8,845 kg), the odds ratio for increasing the rotor diameter is less than 1, including the upper bound of the 95% confidence interval. This result indicates that within this MTOW range, it is better from a safety perspective to increase the rotor diameter. No statistically significant results can be ascertained outside this range given that the 95% confidence interval include the null value OR = 1.

Again, the causal basis for this intriguing result cannot be resolved with a statistical analysis such as this one, but the data and the modeling clearly demonstrate an impact of varying the rotor diameter on the likelihood of helicopter accidents. The results are in some ways almost the opposite for single- and twin-engine turboshaft helicopters. While this deserves careful attention and further engineering analyses, there are some meaningful implications that can help inform the design of future helicopters.

### 3.3 Limitations

It is important to acknowledge the limitations of any statistical analysis, especially with observational studies such as the retrospective cohort study undertaken in this work. The most important limitation is that none of the results derived here should be taken as causal in nature. We established important relationships between the different covariates and the response variable, namely the likelihood of accidents, but these are merely associations, not causal in nature. The causal pathway, if it exists, may be mediated by several other things not accounted for here, and it cannot be ascertained in observational studies. The other limitation concerns the absence of two particular covariates in this study, namely the helicopter usage or mission type, and the intensity of usage or flight hours. Some of our results are possibly confounded by these missing variables.

Mission type, such as helicopters use for law enforcement, local news gathering, sight-seeing tours, or emergency medical services (HEMS), can modify the risk exposure of a rotorcraft to accidents beyond its particular configuration. For example, the 6B heavy twin-turboshaft helicopters with one of the highest (worst) probability of accidents are used for external-load operations, and this distinctive usage may account for its safety track-record. The difficulties in accounting for this usage variable are twofold: first, the FAA does not collect this data except for a small voluntary survey of operators, and when collected, the helicopters in question are not classified according to our covariates (no number of main rotor blades for example); second, it is likely that some helicopters are put to multiple use, or fractional use for different mission types. The absence of mission type data makes this variable unsuitable for inclusion in statistical analysis.

The absence of flight-hours should also be acknowledged as a limitation in this work. This requires some detailed explanation. It was addressed in a previous work by Churchwell et al. [1], and it is worth repeating here for ease of reading and to keep this work self-contained. Limited helicopter flight-hours data are collected in the US. Several authors, and the NTSB have lamented this state of affairs. For example, Fox [20] stated that “the lack of flight-hour exposure data [is a major] roadblock to the helicopter industry. If we cannot measure risk, we cannot tell whether our “improvement” is an actual one or whether it made the problem worse”. Knetch and Smith [21] recognized this limitation as well in their study of training school and examiner type on general aviation flight safety. The authors acknowledged that, “some readers will be disappointed and would prefer to see a study based on, say, accident per flight-hour, or per departure. Unfortunately, that kind of [exposure] data is not readily available.” The NTSB has also been cognizant of this limitation, in the case of helicopter medical emergency services for example. A snippet from the letter of a NTSB Board member is worth quoting: “I am concerned that the [voluntary survey] will not yield the information required to adequately monitor and evaluate the safety record of the EMS industry” [11]. What is the issue referred to here? The FAA estimates the flight hours of helicopters based on a voluntary annual survey known as the “General aviation and Part 135 Activity Survey”. The reluctance of the previously quoted authors to use this data is due to in part to the inherent voluntary response bias in this sampling exercise. We were not able to use these FAA flight-hour estimates in this work for some additional reasons:

1. The survey lumps different configurations of helicopters together (for example, all twin-engine turboshaft helicopters into a single category), and a single estimate of the flight hours for each category of helicopter is provided. This is not appropriate since the 2B-and-3B, the 4B, the 5B, and the 6B helicopters can be found with twin engines, and these helicopters can have different intensity of use. As a result, the estimate provided likely aggregates highly different (multi-modal) data points, and for which the mean is an otiose statistic. Furthermore, the bias in this estimate can be aggravated if for example the response rates are different for operators of the four-bladed helicopters than, say, the six-bladed ones. Since no such information is available, it is best to treat the survey results with guarded skepticism;
2. More importantly, since we were interested in examining, among other things, whether differences in number of main rotor blades are associated with different probability of accidents, the absence of this classification in the FAA estimates renders them unusable for our purposes.

The possibility that these missing variables confound our results can be mitigated to some extent by comparing only configurations that are likely to be used for similar mission types and with comparable intensity of usage. We conclude this subsection by noting that better data collection is warranted and recommend that the FAA collect measures of exposure for rotorcraft (e.g., flight-hours, usage) stratified appropriately in order to enable calculations of accident rates for different helicopter configurations. We reiterate what was noted earlier, that “the lack of flight-hour exposure data [is a major] roadblock to the helicopter industry. If we cannot measure risk [accident rates], we cannot tell whether our “improvement” is an actual one or whether it made the problem worse”.

## 4. Conclusions

In this work, we demonstrated the power of logistic regression to model turboshaft helicopter accidents. Our objective was to advance the understanding of these accidents, and to identify important issues for different stakeholders in the rotorcraft community to focus their attention on for accident prevention. This work, as noted in the Introduction, is part of a larger effort whose end-objective is to contribute toward improving the safety track record of helicopters, and ultimately to reduce the burden of injuries, fatalities, and financial losses due to helicopter accidents.

After a careful model-building process, we determined that four covariates, the number of main rotor blades, the number of engines, the rotor diameter, and the MTOW, are associated with the response variable of the model, namely the probability of accidents. Furthermore, we found that three interactions between these covariates are important and statistically meaningful. They are the interactions between the number of main rotor blades and the number of engines; the number of engines and the rotor diameter; and the rotor diameter and the MTOW. Then, having established and assessed the goodness-of-fit of the model, we leveraged it for inferential purposes. More specifically, we examined the adjusted odds ratio of accidents by changing one covariate at a time, and we teased out important implications in the process. Some of the important questions we addressed are the following

Q1. Do turboshaft helicopters with different number of main rotor blades have different odds of accidents, and if so, are these differences statistically significant? The answers to this are nuanced and required careful explanation. The answer to Q1 depends on the number of engines: for single-engine turboshaft helicopters, the four-bladed ones outperform (lowest odds of accidents) all the others in a statistically significant way. Whereas for twin-engines, no statistically significant difference is detected in the odds of accidents by varying the number of main rotor blades and holding all else constant.
Q2. Do twin-engine turboshaft helicopters exhibit a safety advantage over their single-engine counterparts (all else being equal), and, are these differences statistically significant? The results are more involved than what the common-sense answer would have been expected to be. We found that no one-size-fit all answer exists, and that the results are contingent on both the number of main rotor blades and the rotor diameter. More specifically, we found that (1) the adjusted odds ratio (twin- versus single-engine) of accidents increases (worsens) as the rotor diameter increases; (2) a tipping point exists beyond which twin-engine turboshaft helicopters have a higher propensity to accidents than the single-engine ones, all else being equal; (3) there are regions within the range of the rotor diameter below or above which the differences in the odds are statistically significant.
Q3. Is it better, worse, or irrelevant from a safety perspective to increase the main rotor diameter, all else being equal? What we found was that the answer is contingent on the number of engines and the MTOW. For example, for twin-engine turboshaft helicopters, there was an increase in the propensity to accidents along with an increase in rotor diameter, although there are regions of rotor diameter where the odds difference is not statistically significant. We causally summarized this finding by stating that for twin-engine turboshaft helicopters, it is better to keep the rotor diameter as short as required by other structural and aerodynamic considerations and not any longer. The results, however, were quite different for single-engine helicopters: we found that within a range of 9,000 lbs. (4,082 kg) < MTOW < 19,500 lbs. (8,845 kg), it is better from a safety perspective to increase the rotor diameter since doing so decreases the propensity to accidents.

The results provided here are relevant for different stakeholders in the rotorcraft community, and they lend themselves to a rich set of interpretive possibilities. The causal basis for these results cannot be resolved with a statistical analysis such as the one carried out here. Because of their significant safety implications, they deserve nonetheless careful attention from helicopter manufacturers, operators, and regulators.

## Supporting information

S1 Appendix H-L goodness-of-fit table, estimated covariance matrix of fitted model, and the adjusted odds ratio of accidents for twin- versus single-engine. (PDF)
