# Supplementary material for: Statistical learning for turboshaft helicopter accidents using logistic regression
Source: PLoS One. 2020 Jan 13;15(1):e0227334. doi: 10.1371/journal.pone.0227334 (PMC6957302; doi:10.1371/journal.pone.0227334)
Supplement: S1 Appendix — (PDF) [file pone.0227334.s001.pdf]

S1 Appendix

Table A. Observed (Obs) and estimated expected (Exp) frequencies within each group classified by Hosmer-Lemeshow to test goodness-of-fit of the fitted model in Table 5.

		accident = 1		accident = 0		
Group	Prob	Obs	Exp	Obs	Exp	Total
1	0.0503	45	48.3	1263	1259.7	1308
2	0.0556	29	24.1	415	419.9	444
3	0.0733	57	57.4	823	822.6	880
4	0.0787	67	65.9	777	778.1	844
5	0.1057	56	67	665	654	721
6	0.1208	154	147.6	1075	1081.4	1229
7	0.1268	251	265.6	1849	1834.4	2100
8	0.2302	166	149.2	646	662.8	812

Table B. Corresponding estimated covariance matrix of the estimated coefficients in Table 5 for the final model of accidents among turboshaft helicopters.

	$x_{1,1}$	$x_{1,2}$	$x_{1,3}$	x_2	x_3	x_4	x_5	$x_{6,1}$	$x_{6,2}$	$x_{6,3}$	x_7	x_8	x_9	**const.**
$x_{1,1}$	0.0140													
$x_{1,2}$	0.0022	0.0598												
$x_{1,3}$	0.0043	0.0401	0.1997											
x_2	0.0154	-0.0308	-0.0295	0.8780										
x_3	-0.0008	0.0169	0.0123	-0.0276	0.0071									
x_4	-0.0104	0.0659	0.0355	-0.1915	0.0326	0.2210								
x_5	0.0003	-0.0019	-0.0007	0.0059	-0.0010	-0.0090	0.0005							
$x_{6,1}$	-0.0132	0.0104	0.0091	-0.1402	0.0052	0.0284	-0.0011	0.0922						
$x_{6,2}$	-0.0031	-0.0354	-0.0194	-0.0467	-0.0068	-0.0176	0.0003	0.0504	0.2001					
$x_{6,3}$	-0.0021	-0.0089	-0.1378	0.0810	-0.0078	-0.0305	0.0007	0.0462	0.0876	0.7135				
x_7	-0.0003	0.0007	0.0008	-0.0197	0.0007	0.0046	-0.0001	0.0019	-0.0001	-0.0033	0.0005			
x_8	0.0002	-0.0022	-0.0014	0.0050	-0.0010	-0.0055	0.0002	-0.0007	0.0007	0.0015	-0.0001	0.0002		
x_9	-7.E-06	6.E-05	3.E-05	-2.E-04	3.E-05	2.E-04	-9.E-06	2.E-05	-2.E-05	-6.E-05	4.E-06	-5.E-06	2.E-07	
**const.**	0.0423	-0.5372	-0.3648	1.0280	-0.2323	-1.2417	0.0430	-0.1827	0.1999	0.1925	-0.0248	0.0349	-0.0011	8.0618

Table C. The estimated odds ratio for twin- versus single-engine turboshaft helicopters.

ROTORDIA (ft.)	2B or 3B		4B		5B		6B	
	$\widehat{OR}$	95% CI	$\widehat{OR}$	95% CI	$\widehat{OR}$	95% CI	$\widehat{OR}$	95% CI
25.00	0.33	0.14-0.78	0.38	0.21-0.71	0.09	0.03-0.27	0.09	0.01-0.58
26.33	0.37	0.16-0.83	0.43	0.24-0.76	0.10	0.04-0.29	0.10	0.02-0.63
27.50	0.41	0.19-0.88	0.47	0.28-0.80	0.11	0.04-0.31	0.11	0.02-0.67
31.75	0.58	0.30-1.11	0.67	0.45-1.01	0.16	0.07-0.40	0.16	0.03-0.88
32.00	0.59	0.31-1.12	0.69	0.46-1.02	0.17	0.07-0.41	0.16	0.03-0.90
33.50	0.67	0.37-1.22	0.78	0.54-1.12	0.19	0.08-0.45	0.18	0.03-0.99
34.50	0.73	0.41-1.30	0.84	0.59-1.20	0.21	0.09-0.48	0.20	0.04-1.06
35.50	0.79	0.45-1.38	0.92	0.65-1.29	0.22	0.10-0.51	0.21	0.04-1.14
36.00	0.82	0.48-1.43	0.95	0.68-1.34	0.23	0.10-0.53	0.22	0.04-1.18
37.00	0.90	0.52-1.53	1.04	0.74-1.45	0.25	0.11-0.57	0.24	0.05-1.26
39.33	1.09	0.65-1.81	1.26	0.89-1.78	0.31	0.14-0.68	0.29	0.06-1.49
42.00	1.36	0.82-2.25	1.57	1.06-2.33	0.38	0.17-0.84	0.37	0.07-1.82
44.00	1.60	0.95-2.69	1.85	1.19-2.90	0.45	0.20-1.00	0.43	0.09-2.13
45.25	1.78	1.04-3.03	2.06	1.27-3.33	0.50	0.22-1.12	0.48	0.10-2.35
46.00	1.89	1.10-3.26	2.19	1.32-3.63	0.53	0.24-1.20	0.51	0.11-2.49
48.00	2.23	1.25-3.99	2.58	1.464.58	0.63	0.27-1.45	0.61	0.12-2.94
48.25	2.28	1.26-4.10	2.64	1.47-4.72	0.64	0.28-1.49	0.62	0.13-3.00
49.21	2.47	1.34-4.54	2.86	1.54-5.29	0.70	0.30-1.64	0.67	0.14-3.25
50.00	2.63	1.40-4.94	3.05	1.60-5.81	0.74	0.31-1.77	0.71	0.15-3.47
53.00	3.38	1.65-6.90	3.91	1.838.34	0.95	0.38-2.42	0.92	0.19-4.51
54.50	3.82	1.79-8.18	4.43	1.9610.02	1.08	0.41-2.84	1.04	0.21-5.16
56.00	4.33	1.93-9.74	5.02	2.0912.03	1.22	0.45-3.34	1.18	0.23-5.91
60.00	6.03	2.33-15.61	6.99	2.48-19.68	1.70	0.56-5.22	1.64	0.31-8.61
62.00	7.12	2.56-19.84	8.25	2.70-25.20	2.01	0.62-6.56	1.93	0.36-10.46
69.88	13.69	3.62-51.67	15.85	3.75-67.06	3.86	0.90-16.48	3.71	0.59-23.28
72.00	16.32	3.97-67.08	18.91	4.09-87.41	4.61	1.00-21.24	4.43	0.67-29.14
